# Preliminary study on the mechanisms of ankle injuries under falling and impact conditions based on the THUMS model

**DOI:** 10.1080/20961790.2021.1875582

**Published:** 2021-05-20

**Authors:** Zhengdong Li, Jianhua Zhang, Jinming Wang, Ping Huang, Donghua Zou, Yijiu Chen

**Affiliations:** a Department of Forensic Medicine, School of Basic Medical Sciences, Fudan University, Shanghai, China; b Shanghai Key laboratory of Forensic Medicine, Academy of Forensic Science, Ministry of Justice, Shanghai, China

**Keywords:** Forensic sciences, ankle injuries, fall and impact injury, finite element analysis, injury biomechanisms

## Abstract

Ankle injuries are common in forensic practice, which are mainly caused by falling and traffic accidents. Determining the mechanisms and manners of ankle injuries is a critical and challenging problem for forensic experts. The identification of the injury mechanism is still experience-based and strongly subjective. There also lacks systematic research in current practice. In our study, based on the widely used Total Human Model of Safety 4.0 (THUMS 4.0), we utilized the finite element (FE) method to simulate ankle injuries caused by falls from different heights (5 m, 10 m and 20 m) with different landing postures (natural posture, inversion, eversion, plantar-flexion and dorsi-flexion) and injuries caused by impacts from different directions (anterior-posterior, lateral-medial and posterior-anterior) with different speeds (10 m/s, 15 m/s and 20 m/s) at different sites (ankle and lower, middle and upper sections of leg). We compared the injury morphology and analyzed the mechanisms of ankle injuries. The results showed that falling causes a specific compression fracture of the distal tibia, while fractures of the tibia and fibula diaphysis and ligament injuries caused by falling from a lower height or inversion, planter flexion or dorsiflexion at a large angle are not distinguishable from the similar injury patterns caused by impact on the middle and upper segments of the leg. No obvious compression fracture of the tibia distal was caused by the impacts, whereas ligament injuries and avulsion fractures of the medial or lateral condyle and fractures of the diaphysis of the tibia and fibula were observed. Systematic studies will be helpful in reconstructing the ankle injury processes and analyzing the mechanisms in forensic practice, providing a deeper understanding of ankle injury mechanisms for forensic experts.

## Introduction

Ankle fractures are among the most common intra-articular fractures [1,2] and are mainly caused by falls, sprains and traffic accidents. Due to the different force loading sites, directions, magnitudes and movement states of the lower limb, the injury sites, degrees and morphologies vary significantly, resulting in extremely complicated and diverse mechanisms of ankle injuries. However, the current identification approaches on ankle injury mechanisms are still mainly relied on expertise-based ana­lysis [3,4], which does not facilitate objective judgments and is insufficient for the objective requirement of forensic evidence.

The finite element (FE) method has been widely employed in the fields of biomechanics and bioengineering. It also has been introduced into the field of life sciences and other interdisciplinary subjects, making an outstanding contribution to the clinical analysis of biomechanisms and the comparison of clinical treatment methods [5–7]. Numerous FE models have been established to analyze vehicle safety performance and explore the biomechanisms of ankle injuries in traffic accidents [8,9].

In the 1990s, scholars established FE models of the foot and ankle for research on vehicle safety protection, but the models were simplified with low bionics. In 2005, Iwamoto *et al*. [10] improved the constitutive models of the tibia cortex in the lower extremity model, making it more realistic and more accurate in simulating fractures. A series of dummies and models were used to explore the injury mechanism of ankle under impact, dorsiflexion, axial rotation and complex loads [11–15]. In 2012, Shin et al. [16] established an FE model for simulating car crashes. In 2013, Untaroiu *et al.* [17] used the validated calf-ankle model with a 50th percentile adult size to simulate and observe the ligament ruptures, fractures of the ankle and subtalar joint under different frontal collision loads. The results revealed that ankles with dorsiflexion and inversion could present a similar pedal injury pattern under axial loads. Ankle fractures would occur commonly with small flexion or inversion angles under heavy axial loads on the tibia, while ankle ligament injuries tend to occur with a large inversion or dorsiflexion angle under lighter axial loads. In 2017, Smolen and Quenneville [18] established an ankle FE model to evaluate the bone surface strains in the five most vulnerable postures and finally predicted the fracture thresholds and fracture sites under compression loads, showing the higher risk of talus fracture in natural, eversion-extorsion and planter flexion posture, while frequent fractures of the tibia and fibula in the inversion posture.

As the quantitative and systematic studies on ankle injury mechanisms using the FE method are not common in the published literature, we systematically simulate the ankle injury processes caused by different falling and impact loading conditions by employing the Total Human Model of Safety 4.0 (THUMS 4.0) model, to explore the injury mechanisms and patterns of the ankle injuries, and to assess the value of FE methods in forensic practices.

## Methods

### THUMS 4.0 FE model

**Figure 1.**
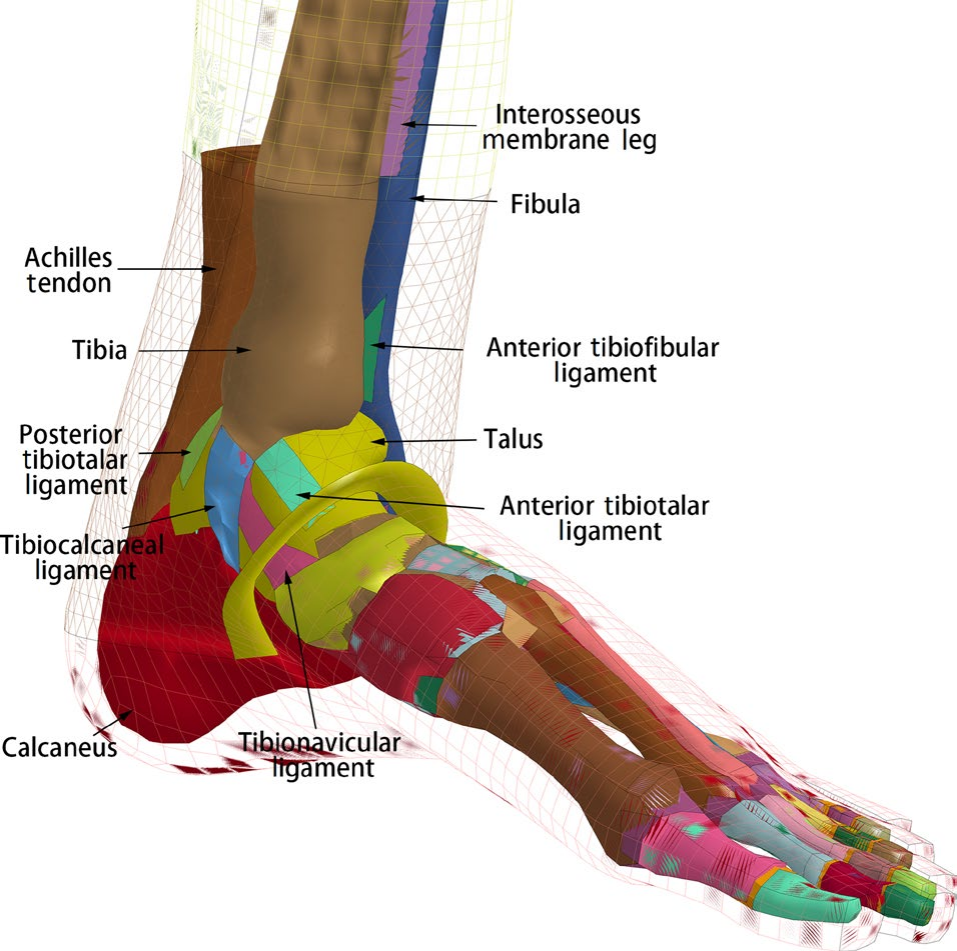
Ankle model of the Total Human Model of Safety 4.0 (THUMS 4.0).

The employed THUMS 4.0 model [19] is a 50th percentile adult male size model, with a height of 175 cm and a weight of 77 kg, which incorporates the main internal organs, vessels, muscle groups and the connective tissue around them. The ligaments in the ankle are modelled with shell elements, while those in the foot are modelled with 1D elements. The Achilles tendon is modelled with solid elements and attached to the calcaneus (Figure 1). All the component models of lower extremity were validated by simulating series of impact tests. The femur is validated by static 3-point bending test performed by Yamada and Evean [20]. The knee validations were dynamic lateral loading performed by Kajzer *et al.* [21] and 4-point bending test conducted by Bose *et al.* [22]. The tibia validation model against dynamic 3-point bending test was conducted by Schreiber *et al.* [23]. The most concerned ankle and foot model is validated by the dynamic axial load test [24].

### Hardware and software

Five HP Z820 Servers (Intel Xeon E5-2687W 3.10 GHz, Nvidia quadro 4000, RAM 64 G, Windows server2008 R2, https://www8.hp.com/h20195/v2/GetPDF.aspx/c04111526.pdf), the LS-DYNA FE solver software (LST, Livermore, CA, USA) and LS-PrePost software (LST) were used.

### Loading conditions

#### Falling loading conditions

We established a ground FE mode by using rigid material and initially set the THUMS model as standing posture. The global gravity acceleration was set as 9.8 m/s^2^. The contact type between the sole and ground was set as CONTACT_AUTOMATIC_SURFACE_TO_SURFACE, with a dynamic friction coefficient of 0.75. We calculated the initial landing speeds of humans according to the principle of free-fall motion (ignoring air resistance and other factors) and loaded the initial parameter on the THUMS model. We set the tilt angle of the ground to simulate the effect of different human foot and ankle postures when making contact with the ground (Table 1).

**Table 1.** Simulation loading condition of ankle injury caused by falling.

Variables	Parameters
Fall height	5 m, 10 m, 20 m
Ankle posture	Planter flexion, dorsiflexion, inversion, eversion, natural posture
Posture angle	10°, 20°, 30°

#### Impact loading conditions

We established ground model and global gravitational acceleration according to falling conditions and reconstructed a simplified rigid bumper model. We generated a simulation matrix to study the injury biomechanism under series of impact loading conditions, including the different impact sites of the leg (100, 125, 240 and 350 mm above the ground, respectively), different impact directions and different speeds (Table 2). We extracted the deformation, displacement, stress and strain data of different anatomical sites from the simulation results and analyzed the biomechanism of the ankle injuries.

**Table 2.** Simulation loading conditions of ankle injury caused by impact.

Variables	Parameters
Impact position	Ankle, lower part of leg, middle part of leg, upper part of leg
Impact direction	Anterior-posterior (A-P), lateral-medial (L-M), posterior-anterior (P-A)
Impact velocity	10 m/s, 15 m/s, 20 m/s

#### Data analysis

We manually extracted data from the simulation results by LS-PrePost software (LSD) and performed statistical analysis by GraphPad Prism 8 (GraphPad Software Inc., San Diego, CA, USA) and SPSS 20.0 (IBM Corp., Armonk, NY, USA).

## Results

### Simulation results under falling conditions with different postures

**Figure 2.**
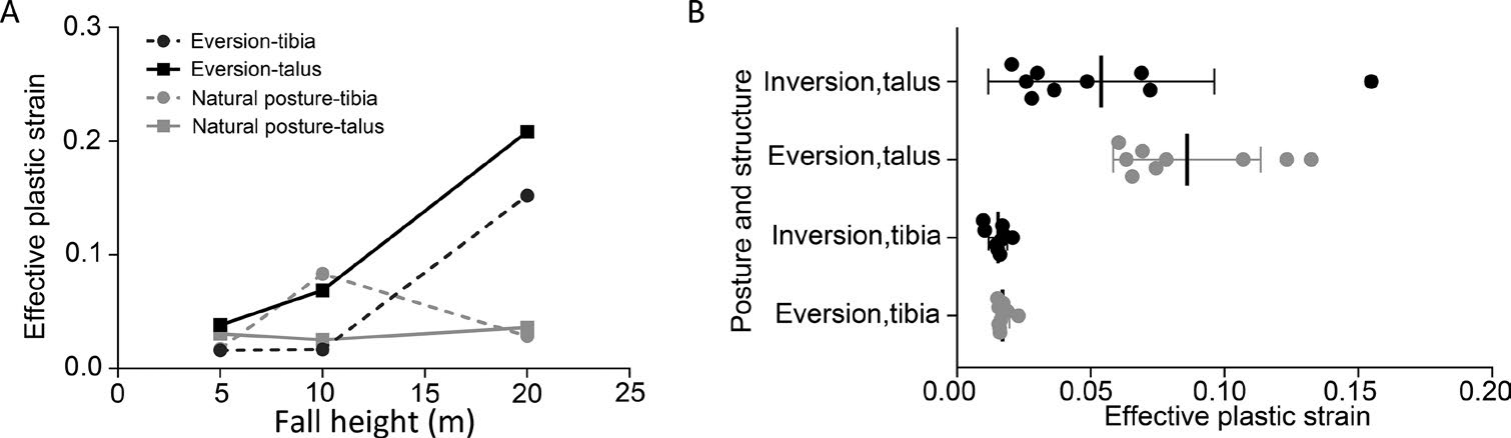
(A) The effective plastic strain of the tibia and talus increases as the height of falling increases, which differs from the natural posture. (B) The distal tibia and talus endure smaller effective plastic strains in inversion posture which may cause less severe injury than eversion posture.

After falling in a natural position, the talus moves outward, backward and upward, leading to compression and fractures of the distal tibia and lateral condyle. Moreover, a stress concentration area in the talus predicts the risk of fracture. The outward displacement of the talus causes extensions in the anterior medial group ligaments (anterior tibiotalar ligament and tibionavicular ligament) and pushes the tibia inward, subsequently increasing the stress on the tibiofibular ligament. The relative downward movement of the tibia and fibula results in a concentration of stress and strain in the posterior talofibular ligament. As the fall height increases, the stress and strain peaks of ligaments do not increase accordingly, but the peaks appear in advance (Table 3). The injury patterns and mechanisms of the ankle in the eversion posture after landing are similar to those in the natural posture. The injury patterns are consistent under all loads in our test. The effective plastic strain of the tibia and talus increases with the height of falling, which differs from the natural posture (Figure 2A).

**Table 3.** Peak strain of main structures when ankle with natural posture after landing.

Fall height (m)	Lateral condyle		Tibia distal		Talus		Medial ligament		Lateral ligament	
	Max	T (ms)	Max	T (ms)	Max	T (ms)	Max	T (ms)	Max	T (ms)
5	1.92e-2	10.2	1.69e-2	11.9	8.84e-2	12.8	0.139	4.0	0.858	4.1
10	1.80e-2	6.5	1.75e-2	6.5	7.45e-2	10.1	0.247	2.8	0.487	2.8
20	1.72e-2	4.3	1.69e-2	4.6	6.71e-1	8.1	0.165	1.8	1.147	1.8

Note: the strains of the bones refer to the effective plastic strain, while strains of ligaments refer to the maximum principal strain. T: time.

**Figure 3.**
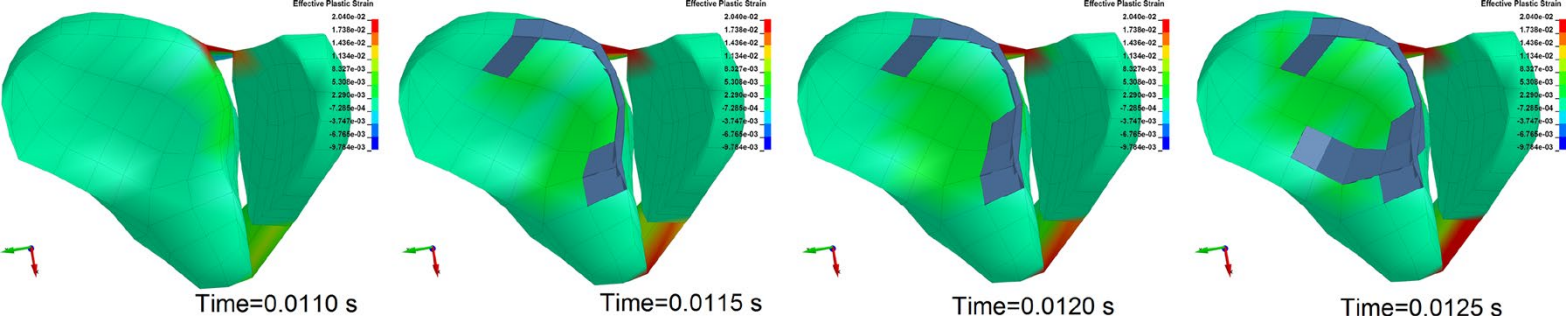
Antapical view of the ankle articular surface (with dorsiflexion of 20° falling from 5 m). The lateral anterior part of the distal tibia fractured first where the effective plastic strain initially increased when ankle shows dorsiflexion after landing.

**Figure 4.**
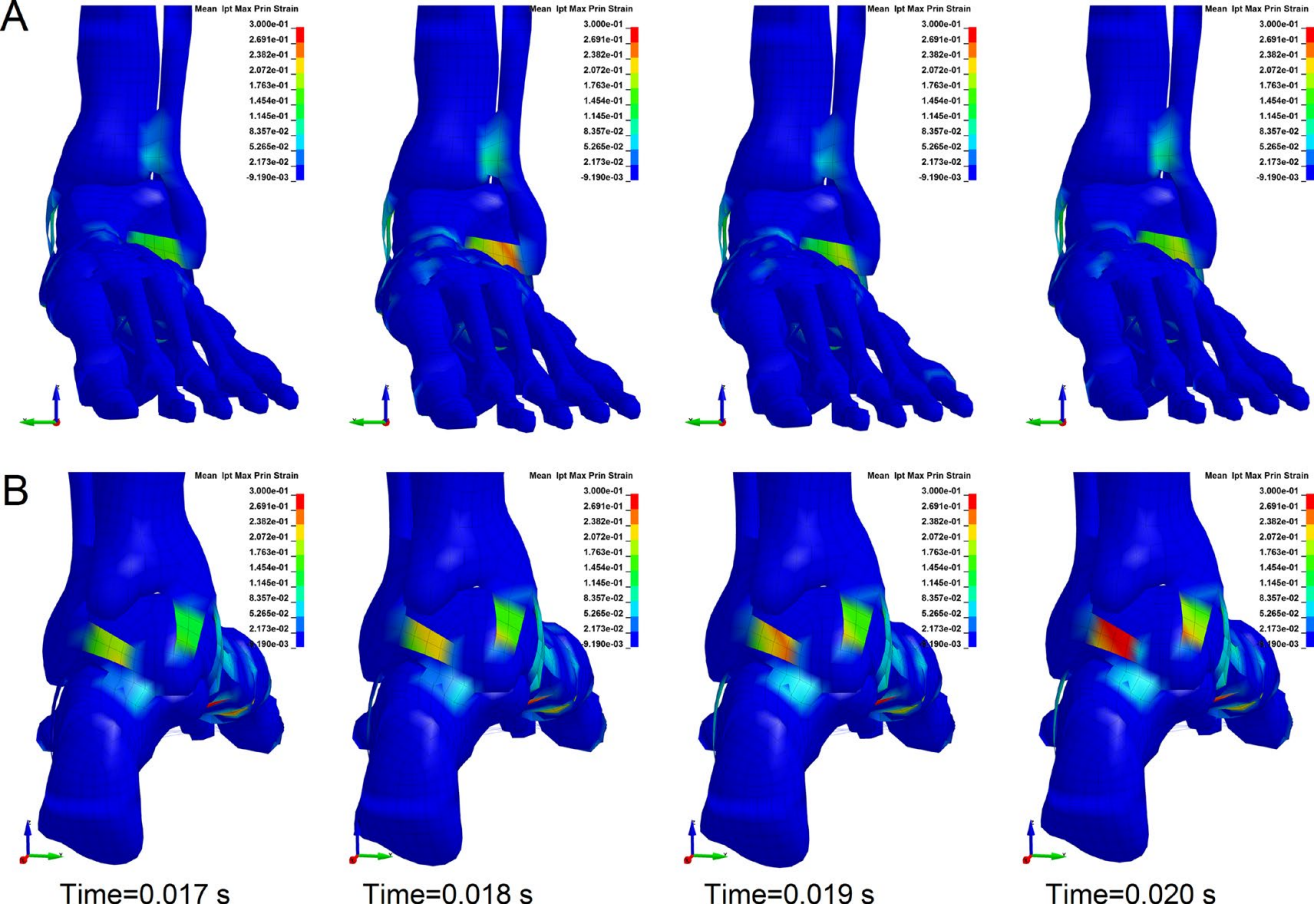
Maximum principal strain contours of the ligaments (ankle with dorsiflexion of 30° falling from 5 m). (A) Anterior aspect. The maximum principal stress of the anterior talofibular ligament and anterior tibiotalar ligament appears an obvious increase, indicating a high injury risk. (B) Posterior aspect. The maximum principal stress of posterior talofibular ligament and posterior ligament appears an obvious increase, indicating a high injury risk. (Each column is at the same time point).

When the ankle inverses, dorsiflexes and planter flexes after falling to the ground, heights and postures will have significant effects on the injury patterns. When the ankle inverses after landing, the effective stress and effective plastic strain of the lateral condyle and the distal tibia increase as the angle decreases. According to our results, the distal tibia and talus endure smaller effective plastic strains in the ankle with inversion than in the ankle with eversion (Figure 2B). Similarly, the injury patterns are affected by falling height when the ankle planter flexes after landing. It is not easy to generate an intra-articular fracture with a height of 5 m, while compression fractures of the distal tibia are directly generated with heights of 10 and 20 m (Table 4). As the height increases, the effective plastic strain of the tibia and talus increases. The results show that if the ankle dorsiflexes after landing, the lateral anterior tibia articular surface will be compressed and fractured firstly, then the lateral condyle fracture is caused by tibiofibular ligament (TFL) pulling and calcaneus compression (Figure 3). However, only when the fall height is lower (5 m) and the dorsiflexion angle is larger (30°) can the feet and the ground experience relative sliding with no intra-articular fracture; such injuries are characteri­zed by tension and injuries of the posterior and lateral group ligaments (Figure 4).

**Table 4.** Fracture sites of ankle with planter flexion angle after landing.

		Planter flexion angle	
Fall height (m)	10°	20°	30°
5	Middle and lower part of fibula and tibia	Lower 1/3 part of fibula and tibia	Foot sliding continuously without fracture
10	Lateral condyle; anterior and posterior distal of tibia (compression fracture)	Lateral condyle; anterior distal of tibia (compression fracture); medial condyle (avulsion fracture)	Lateral condyle; medial condyle (avulsion fracture)
20	Lateral condyle; anterior distal of tibia (compression fracture)	Anterior distal of tibia (compression fracture); medial condyle (avulsion fracture)	Lateral condyle; anterior distal of tibia (compression fracture)

For the complex postures, the ankle fractured only when the ankle had dorsiflexion and inversion of 10° and dorsiflexion and eversion of 10° in the loading conditions, in which the position of the ankle was relatively fixed. However, there is no intra-articular fracture in the ankle with planter flexion and inversion of 10° or planter flexion and eversion of 10°. Detailed injury simulation results are shown in Table 5.

**Table 5.** Injury patterns of ankle with composite postures after landing.

Posture	Change of posture	Fracture site	Ligament (strain increased)
Planter flexion and inversion of 10°	Feet slip outward caused extorsion ankle	Middle part of the tibia and fibula	Posterior talofibular ligament, posterior tibiofibular ligament
Planter flexion and eversion of 10°	Hell slip downward caused extorsion ankle	Middle part of the tibia and fibula	Medial and lateral group ligament, tibiofibular ligament
Dorsiflexion and inversion of 10°	No slipping	Anterior and lateral distal of tibia; upper lateral condyle (talus possible)	Lateral group ligament; anterior and posterior tibiofibular ligament
Dorsiflexion and eversion of 10°	Heel slip inward make ankle pronate	Lateral distal of tibia; medial condyle; lateral condyle	Lateral group ligament; anterior and posterior tibiofibular ligament

### Results of impact loading conditions

**Figure 5.**
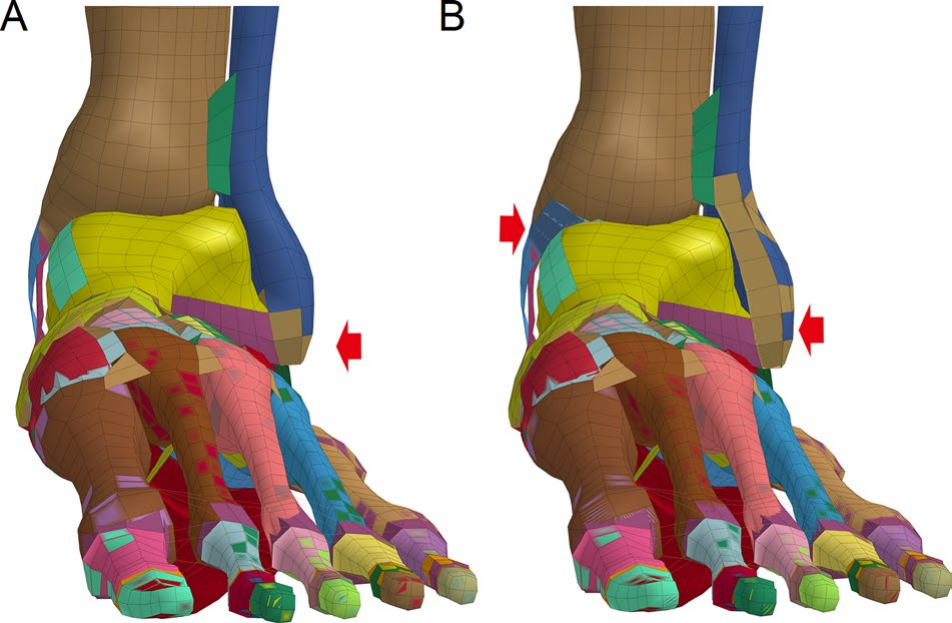
Ankle fractures formed by lateral-medial (L-M) impacts on the ankle. (A) Impacts at lower speeds (10 or 15 m/s) cause lateral condyle fractures. (B) Impacts at higher speeds (≥20 m/s) cause lateral condyle fractures and avulsion fractures of the medial condyle. (Red arrow indicates the fracture site where elements are coloured differently from the surrounding area).

**Figure 6.**
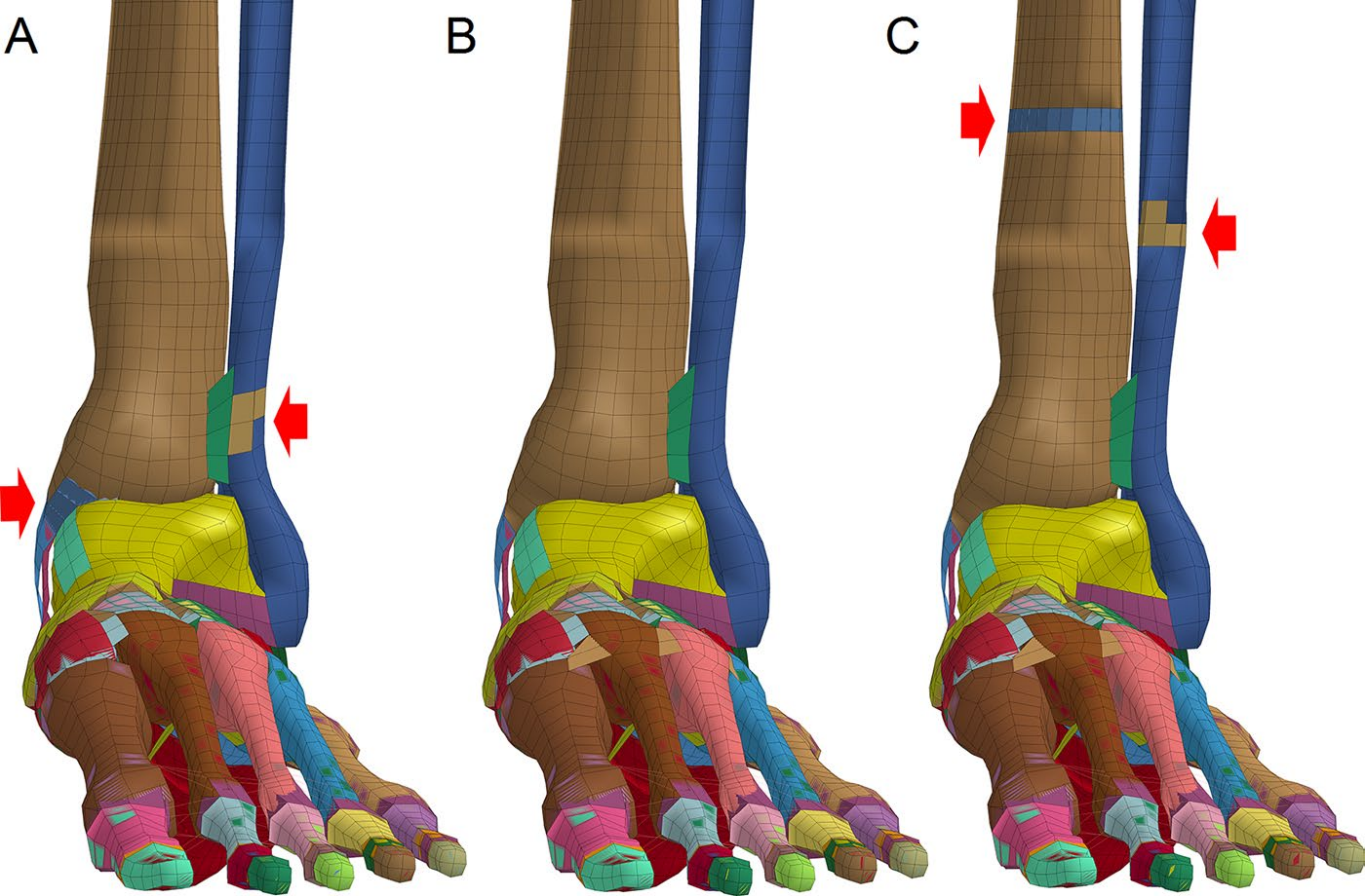
Ankle fractures formed by posterior-anterior (P-A) impacts on the ankle. (A) Impact at a speed of 10 m/s caused lateral condyle fracture and avulsion fractures of the medial condyle. (B) Impact at a speed of 15 m/s did not cause fractures. (C) Impact at a speed of 20 m/s caused tibia and fibula fractures at the impact points. (Red arrow indicates the fracture site where elements are colored differently from the surrounding area).

The anterior-posterior (A-P) impacts on the ankle mainly cause anterior medial ligament (anterior tibio­talar ligament and tibionavicular ligament) injuries but generally do not cause a fracture, and no intra-articular fractures were formed under all speeds. The effective strains of ankle bones and ligaments increase with increasing impact speeds (Table 6). Lateral-medial (L-M) impacts on the ankle directly cause lateral condyle fractures, leading to outward rotation of the ankle with extorsion. In addition, avulsion fractures generated at high impact speeds (≥20 m/s) are different from fracture morphologies under low-speed lateral impacts (Figure 5). Posterior-anterior (P-A) impacts with lower speeds (10 or 15 m/s) on the ankle cause stress concentrations at impact sites, knee extension and medial and lateral ligament extensions around the ankle. However, lateral condyle fracture and avulsion fracture on the medial condyle formed after impacts with a speed of 10 m/s, while impact at 15 m/s did not cause fractures. When the impact speed increases (20 m/s), the tibia and fibula bone are fractured at the impact sites, and the posterior medial group ligaments of ankle are obviously stretched (Figure 6).

**Table 6.** Peak strain of main structure of impacted (anterior-posterior, A-P) ankle.

Impact velocity (m/s)	Lateral condyle	Tibia distal	Talus	Lateral ligament	Medial ligament	Intermedia ligament
10	–	1.21e-4	–	0.136	0.120	0.073
15	1.23e-3	1.41e-3	–	0.218	0.195	0.139
20	3.86e-3	3.30e-3	–	0.295	0.260	0.189

Note: the strains of the bones refer to the effective plastic strain, while strains of ligaments refer to the maximum principal strain.

**Figure 7.**
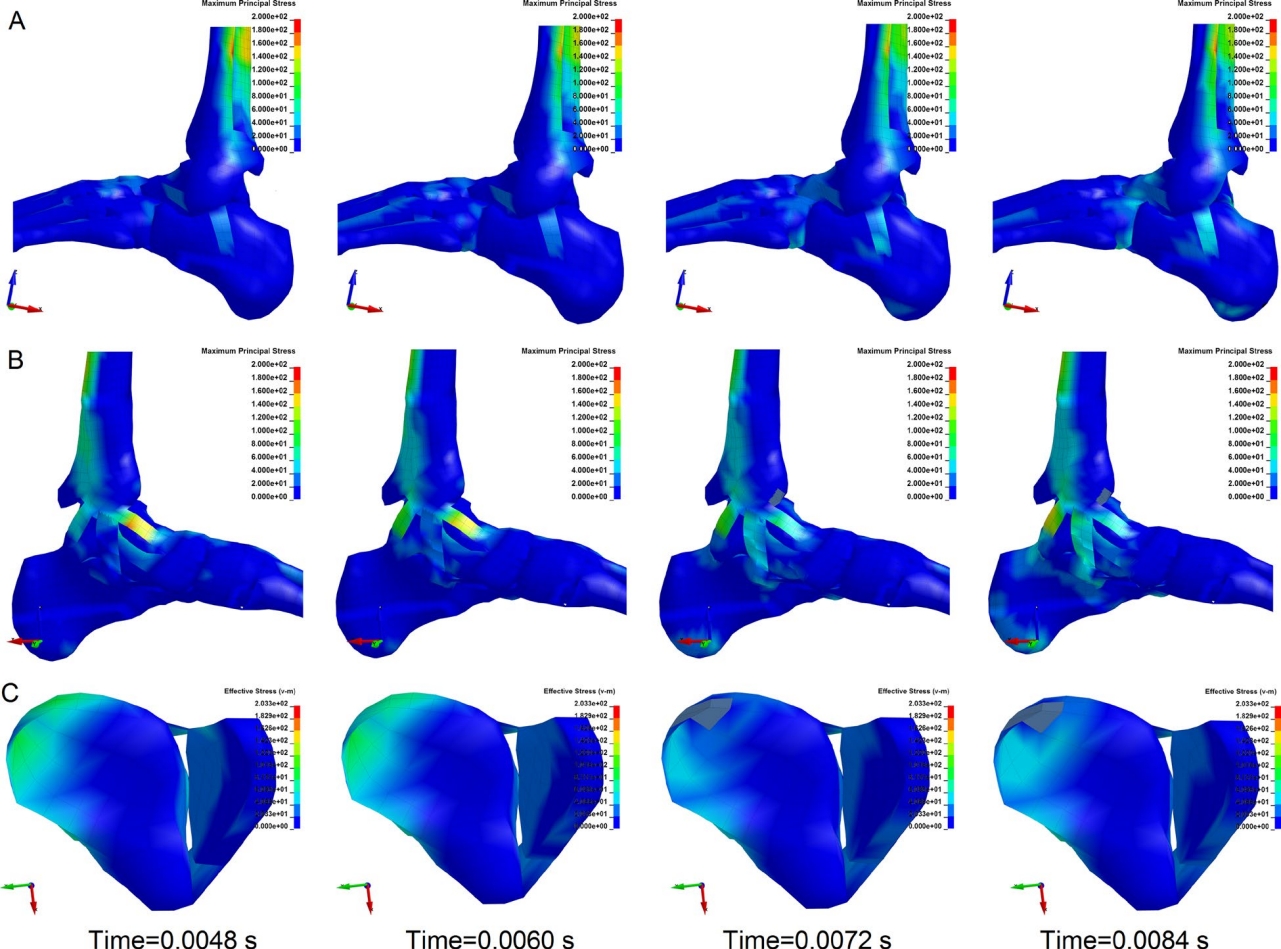
Stress contours of ligaments and tibia (anterior-posterior, A-P impact on lower leg). (A) Contour of lateral ligaments’ maximum principal stress. Maximum principal stress increased in the talofibular ligament. (B) Contour of medial ligaments’ maximum principal stress. Maximum principal stress of the anterior deltoid ligament obviously increased, indicating a high risk of injury. (C) Contour of effective stress in the distal tibia and fibula. Effective stress of the medial part of the distal tibia, where pulling by anterior deltoid ligament increased and caused avulsion fracture. (Each column is at the same time point).

**Figure 8.**
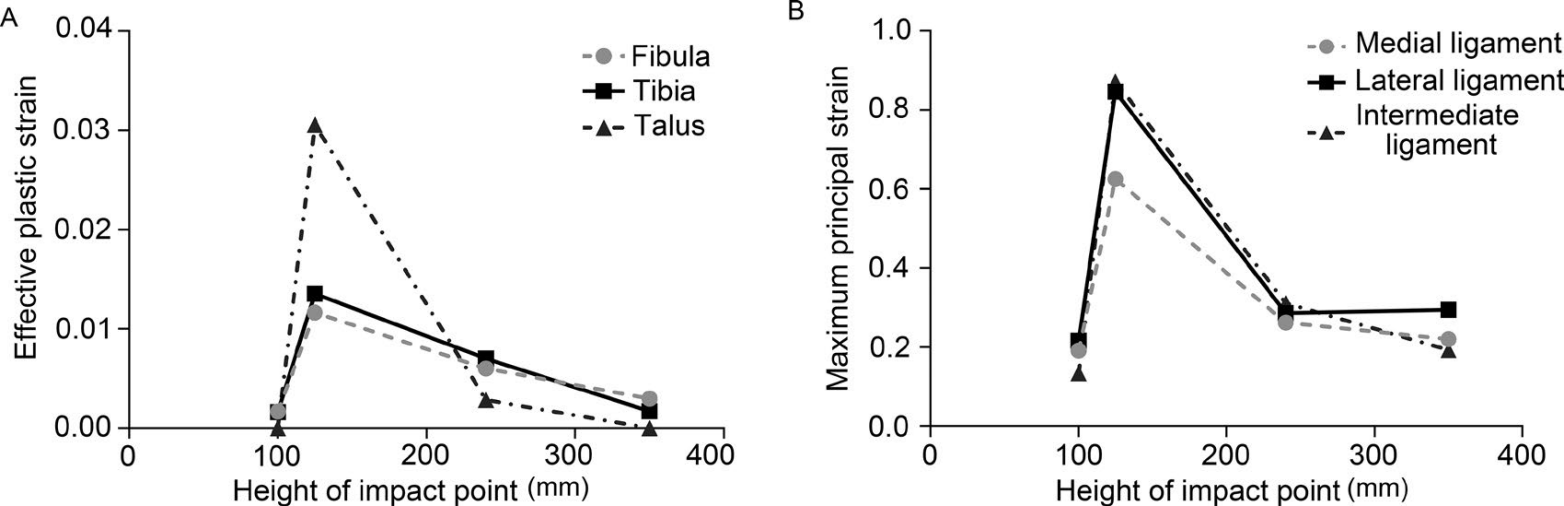
Strains of the main structures of ankle caused by anterior-posterior (A-P) impacts on points with different heights. (A) The maximal bone effective plastic strain caused by A-P impacts on the lower leg (height of 125 mm). (B) The peak ligaments’ maximum principal strain was also caused by impacts on the lower leg, indicating that A-P impacts on the lower leg may result in more serious injuries of both bones and ligaments than A-P impacts on other sites.

**Figure 9.**
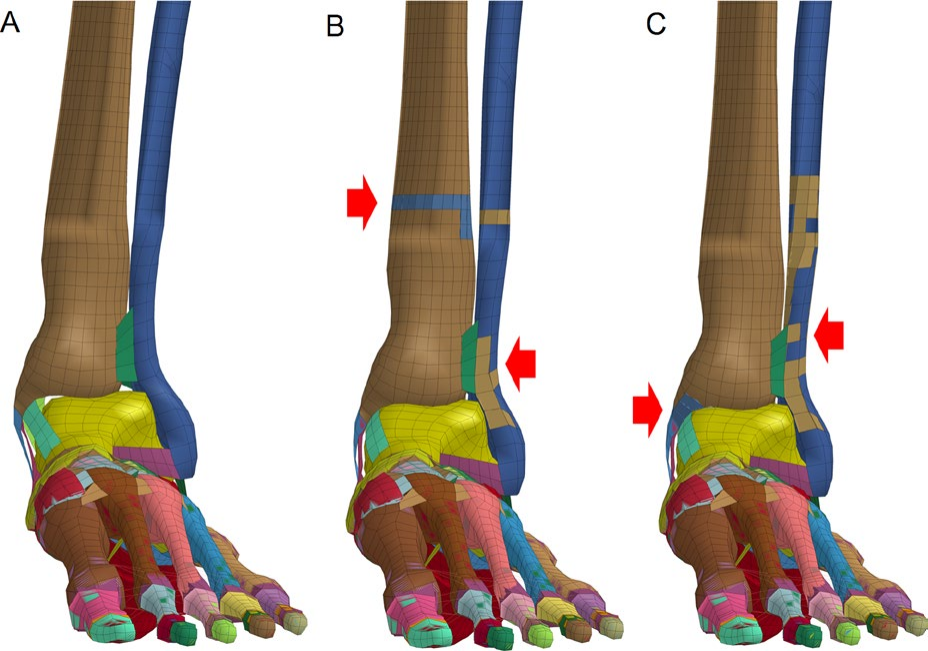
Ankle fractures caused by lateral-medial (L-M) impacts on the lower leg. (A) Impact at a speed of 10 m/s did not cause ankle fractures. (B) Impact at 15 m/s caused fractures of the lateral condyle and impact site. (C) Impact at 20 m/s caused fractures of the lower fibula and avulsion fractures of the medial condyle. (Red arrow indicates the fracture site where elements are coloured differently from the surrounding area).

The injury mechanism of the ankle caused by A-P impacts on the lower leg at different impact speeds is similar to that of impacts on the anterior ankle. The ankle dorsiflexed excessively, and the ligaments of both sides were overstretched, causing an avulsion fracture of the medial condyle. The injury risk of the deltoid ligament and posterior talofibular ligament is great due to the maximum principal stress increase (Figure 7). Compared with the A-P impacts on the ankle and middle and upper segments of the leg, A-P impacts on the lower segment of leg lead to greater stress and strain, which may result in serious injuries of both bones and ligaments (Figure 8). L-M impacts on the lower leg with lower (10 m/s) and higher speeds (15 and 20 m/s) can form different injury patterns (Figure 9). When the lower leg undergoes P-A impacts at lower speeds (10 or 15 m/s), the medial talus will collide with the medial condyle, resulting in an increase of effective stress and effective plastic strain, but no intra-articular fractures are formed. Impact at higher speed (20 m/s) can cause a stress increase and intra-articular fracture of the medial condyle, the posterior tibia distal and the talus.

A-P and P-A impacts on the middle and upper segment of the leg with all speeds lead to fractures of the fibula and tibia at the impact sites. However, the bone’s effective plastic strain in the ankle is smaller. Ankle ligament injuries are mainly caused by impacts with higher speeds. A-P impacts and P-A impacts cause both lateral and medial ligament and calcaneofibular ligament extension, respectively. L-M impacts on the middle segment of the leg formed impact site fractures with higher impact speeds (15 or 20 m/s), while the stresses and strains of ligaments and bones did not increase significantly.

## Discussion

In forensic practices, ankle injury mechanisms are complicated and diversified in fall and traffic accident conditions. The mechanisms and manners of ankle injuries urgently need clarification in civil and criminal disputes [3,4]. At present, research on ankle injury mechanisms remains at the stage of experience judgments. Some scholars [15-17, 20-21] have studied the mechanisms mainly from the perspective of clinical and sports medicine, rather than forensic medicine. Moreover, according to the statistics of forensic cases, pedestrians accounted for 38.5% of ankle injuries caused by traffic accidents [23], but there are few reports on the mechanism of pedestrian ankle injuries. The FE model overcomes the limitations of cadaveric experiments and animal experiments, with the characteristics of low cost, high efficiency and good repeatability. The THUMS 4.0 human FE model was jointly developed with a fine structure, grid quality and sufficient validity verification to clearly reconstruct changes in the human body under loading conditions. We systematically simulated ankle injuries under various loads and summarized the characteristics of the injury to analyze the differences in ankle injury mechanisms caused by falling and impact from the perspective of forensic biomechanics. We summarized the characteristics of the injuries generated by different loading conditions to provide references for identification in forensic cases. According to the Lange-Hanson classification [24] and our experience, falling conditions cause type V ankle fracture, which is a vertical compression fracture. However, based on our simulation results, not every falling condition will result in a compression fracture. It was clear that the conventional view did not take more complex injury conditions encountered in forensic cases into consideration, such as falling heights and ankle postures after landing. According to our results, if the ankle is in a natural posture after landing, the fracture patterns caused by falling are not only consistent with the vertical displacement of the comminuted fracture of the lower extremity described in the Lange-Hanson classification [24] but also in accord with the longitudinal ankle fracture described in the Ashurst-Bromer classification [25]. At the same time, the maximum principal stresses of medial ankle ligaments will usually significantly increase, predicting the risk of ligament rupture. However, our results show that the stresses and strains of ankle ligaments do not always increase when the falling height increased. The distal tibia and the lateral condyle of the fibula will fracture in the eversion postures under all injury loads, with the same patterns and mechanisms found in natural postures. But the strains of tibia and talus increase when in falling height increased in the eversion posture, which may indicate more serious ankle injuries. The typical vertical compression fractures were formed in the inversion posture, except for the load with a lower falling height and inversion of a larger angle. This kind of fracture is also caused by falling from 10 to 20 m when the ankle undergoes planter flexion after landing but is not easily formed when lower falling height. In our study, when the ankle is in a natural posture, it is easy to form a typical compression fracture distal to the tibia and fibula, while the corresponding position of the talus has an increased stress, indicating the fracture risk. When the ankle is in the inversion, planter flexion and dorsiflexion postures after landing, with higher falling heights and smaller angles, typical compression fracture of the tibia and a possible talus fracture will be formed. In contrast, with a lower falling height and larger posture angles, the relative sliding between the foot and the ground will form fractures of the diaphysis of the tibia and fibula, which may not be a simple fracture patterns caused by bending moments. In this study, the rule of ankle injuries caused by falling is closer to Untaroiu et al.’s [17] conclusion obtained in research on the ankle injuries caused by the axial loads due to the pedal impact during car collision. This suggests that in forensic practice, we should consider the falling height and the ankle postures after loading when explaining ankle and leg injuries, especially in the case of an atypical fracture.

At present, there are some FE studies [26–28] on traffic accidents in the field of automobile safety and injury prevention. However, there is few studies on the morphology and mechanism of pedestrian injury. In the field of forensic science, Li *et al.* [29] attempted to use the FE method to distinguish the injuries of the lower limb caused by running over and impact in a real case. Our results show that the A-P impacts lead to the dorsiflexion of the ankle and flexion of the knee, which convert the impact energy into the kinetic energy of the leg effectively, with no fracture formed under all loads, which is determined by the movement of the knee joint. In contrast, P-A impacts will lead to ankle fractures and ligament ruptures due to the limitation of knee joint extension angle resulting in little movement of the ankle and lower leg. P-A impacts can cause different injury morphologies at different speeds. When the ankle encounters L-M impacts, the directly impacted lateral condyle will fracture at lower impact speeds, due to its weak structure and the extorsion with the tibia and talus. The avulsion fractures in the medial condyle are formed when the impact speeds are larger due to traction of the deltoid ligament. The ankles are impacted directly in different directions, and different injury morphologies are formed due to differences in the anatomical structures and magnitudes of load forces.

There are also different injury biomechanisms and morphologies when the ankle is subjected to indirect force. When the lower segment of the leg is hit, the motion states generated by the force in the same direction are similar because the anatomy of the lower leg and the ankle is adjacent. It is worth noting that the A-P impacts on the frontal lower leg lead to fractures of the impact sites and avulsion fractures of the medial condyle. Compared with the impacts to the ankle and other parts of the leg, A-P impacts to the lower leg lead to greater stress and strain rise of ankle ligaments and bones, resulting in more serious injuries. When the L-M impacts on the lower leg, because the simulated impact part is located at the binding site of the tibia and fibula, the low-speed impacts do not typically generate tibia and fibula fractures. With increasing impact speeds, fractures are formed at the upper segment of the impact sites in the lower segment of fibula. The P-A impact load on the lower leg will directly generate fractures of the distal tibia, which differs from the mechanism of fracture in the distal tibia caused by P-A impacts on the posterior ankle. When the impact sites rise to the middle and upper segments of the leg, the A-P and P-A impacts lead to fractures at the impact sites and indirectly formed characteristic ankle ligament injuries.

By summarizing the characteristics of ankle injuries under the aforementioned loads, it is easy to find that the injuries caused by falling are mainly characterized by compression fracture in the distal end of the tibia and the most likely fractures of the upper part of the lateral condyle. The most common injury mechanism is the intense interaction between the tibia and talus. However, with a lower fall height or larger angle of the ankle postures, the foot will slide relative to the ground, causing insufficient interaction between the talus and the tibia. Simultaneously, foot sliding will cause a large bending moment and torsion in the middle and lower segments of the tibia and fibula diaphysis, leading to diaphysis fractures. The simulated ankle injury mechanisms are different from the conventional experience that ankle fractures caused by falling do not necessarily include vertical compression fractures. This suggests that when we analyze the mechanisms of ankle injuries in forensic cases, we should consider the effects of falling heights and ground conditions and pay more attention to the mechanisms of fractures in the tibia and fibula diaphysis.

The ankle impacts cannot form compression fractures in the distal tibia, which is one of the main differences from typical ankle injuries caused by falling. The mechanism of ankle injuries caused by impacts mainly contains fractures on direct impact sites, ankle ligament ruptures and avulsion fractures on the medial condyle or lateral condyle caused by exceeding the limits of ankle flexion, extension and eversion of the ankle. For the impact loading conditions, the farther the impact height is from the ankle, the weaker the effects on ankle injuries. The fractures in the tibia and fibula diaphysis are a feature of injuries caused by impacts on the middle and upper segments of the leg but are not specific. According to our findings, we can identify injuries caused by falling when there is a vertical compression fracture in the distal tibia, but we cannot exclude falling when no vertical compression fracture exists. Fractures of the tibia and fibula diaphysis, ligament injuries and avulsion fractures are not distinctive injury patterns. In forensic practice, we should pay more attention to atypical injury patterns, such as tibia and fibula diaphysis fractures caused by falling and no fracture injury caused by A-P impacts on the ankle.

The preliminary study on the biomechanisms of ankle injuries still have some limitations. Due to the lack of reports on the failure criteria of the ankle ligaments, the talus, calcaneus and other foot bone, the injury thresholds of the ligaments, talus and calcaneus are not set in the THUMS 4.0 foot and ankle model. Therefore, the model is unable to directly reconstruct ligament ruptures and fractures of the talus and calcaneal. The measurement of human material properties is still a long-term task in the improvement of the FE model. In addition, ankle injuries should be simulated under more loading conditions, especially complicated loads, to summarize the ankle injury law in more detail and with greater accuracy. Moreover, the model does not implant muscle units that can contract spontaneously either, so it cannot simulate the effect of muscle contraction in the injury process [30–32]. More importantly, the current THUMS model is based on the traditional FE method, which cannot satisfy the simulations of the most realistic fracture morphologies and cannot simulate the formation and extension of the fracture lines, that is, fractures with special shapes (e.g. wedge fracture, spiral fracture, etc.) cannot be truly reproduced. The solution to this problem still depends on the development of FE technologies [33,34].

## Conclusion

This study presents a preliminary biomechanisms of ankle injuries under different injury loading conditions. When the foot falls onto the ground, the ankle assumes different postures due to different ground conditions. The natural and eversion posture of ankle after landing can easily generate typical compression fractures in the distal tibia and fibula and high fracture risks on corresponding position of the talus. The inversion, planter flexion and dorsiflexion of ankle at a large angle after landing can cause fractures of the tibia and fibula diaphysis, which is not consistent with clinical experience and should attract high attention inforensic practices. The direct impacts on the ankle in the radial directions and the front impacts can hardly cause ankle fractures. On the contrary, impact on the lower leg segment generates more severe ankle injuries. Lateral impacts with different speeds lead to various fractures in the lateral condyle or both lateral and medial condyle. The impacts on the middle and upper leg segments can hardly generate intra-articular fractures, except for the ligament injuries. Our prelimi­nary results provide new approaches and some key opinions in identifying the biomechanisms and manners of ankle injuries, which may be employed in the further study of complex injury mechanisms in forensic practices.
